# The effects of virtual reality training on clinical indices and brain mapping of women with patellofemoral pain: a randomized clinical trial

**DOI:** 10.1186/s12891-021-04785-6

**Published:** 2021-10-25

**Authors:** Naghmeh Ebrahimi, Zahra Rojhani-Shirazi, Amin Kordi Yoosefinejad, Mohammad Nami

**Affiliations:** 1 Student Research Committee, School of Rehabilitation Sciences, Shiraz University of Medical Sciences, Chamran Blvd., Abiverdi 1Street, P.O. Box: 71345-1733, Shiraz, Iran; 2 Department of Physical Therapy, School of Rehabilitation Sciences, Shiraz University of Medical Sciences, Chamran Blvd., Abiverdi 1Street, P.O. Box: 71345-1733, Shiraz, Iran; 3 Rehabilitation Sciences Research Center, Shiraz University of Medical Sciences, Shiraz, Iran; 4 Neuroscience Center, Instituto de Investigaciones Científicas y Servicios de Alta Tecnología (INDICASAT AIP), City of Knowledge, Panama City, 084301103 Panama; 5 Department of Neuroscience, School of Advanced Medical Sciences and Technologies, Shiraz University of Medical Sciences, Shiraz, 71348-14336 Iran; 6 Dana Brain Health Institute, Iranian Neuroscience Society-Fars Chapter, Shiraz, 71364-76172 Iran; 7 Academy of Health, Senses Cultural Foundation, Sacramento, CA 66006 USA; 8 Department of Cognitive Neuroscience, Institute for Cognitive Science Studies (ICSS), Pardis, Tehran, 1658344575 Iran

**Keywords:** Patellofemoral pain, Virtual reality, Balance, EEG

## Abstract

**Background:**

Virtual reality training (VRT) is a new method for the rehabilitation of musculoskeletal impairments. However, the clinical and central effects of VRT have not been investigated in patients with patellofemoral pain (PFP). To comprehensively assess the effects of VRT on clinical indices and brain function, we used a randomized clinical trial based on clinical and brain mapping assessment.

**Methods:**

Twenty-six women with PFP for more than 6 months were randomly allocated to 2 groups: intervention and control. The intervention consisted of lifestyle education + 8 weeks VRT, in 24 sessions each lasting 40 min of training, whereas the control group just received lifestyle education. The balance was the primary outcome and was measured by the modified star excursion balance test. Secondary outcomes included pain, function, quality of life, and brain function which were assessed by visual analogue scale, step down test and Kujala questionnaire, SF-36, and EEG, respectively. Pre-intervention, post-intervention and follow-up (1 month after the end of the intervention) measurements were taken for all outcome measures except EEG, which was evaluated only at pre-intervention and post-intervention). Analyses of variance was used to compare the clinical outcomes between the two groups. The independent t-test also was used for between group EEG analyses.

**Results:**

Balance score (*P < 0.001*), function (*P < 0.001*), and quality of life (*P = 0.001*) improved significantly at post-intervention and 1 month follow-up in the VRT group compared with the control group. VRT group showed a significantly decreased pain score (*P = 0.004*). Alpha (*P < 0.05*) and theta (*P = 0.01*) power activity also increased in the brain of the VRT group.

**Conclusion:**

This study demonstrated that long term VRT was capable of improving both clinical impairments and brain function in patients with PFP. Therefore, therapists and clinicians can use this method as a more holistic approach in the rehabilitation of PFP.

**Trial registration:**

IRCT, IRCT20090831002391N40. Registered 23 / 10 / 2019.

## Background

Patellofemoral pain (PFP) is described as a non-specific pain in the anterior surface of the knee with a multifactorial etiology [1]. The pain is aggravated by activities involving repetitive loading on the patellofemoral joint, such as squatting, stair climbing, running, kneeling, and so on [2]. Incidence of PFP is about 22 per 1000 persons in a year and women are more vulnerable [3]. Prevalence of PFP in Iranian female athletes is 16.74% [4]. Distinct anatomical and biomechanical differences among sexes, such as less cartilage thickness, more peak stress on the cartilage during stair climbing, increased Q-angle, dynamic valgus angle, and hip internal rotation angle in women can cause more incidence and prevalence of PFP in women [5].

Postural control can be impaired in patients with PFP [6–8]. Muscle weakness, pain, abnormal motor control, and impairment in proprioception inputs are possible reasons for the alteration of postural control in these patients [6, 9]. Pain threshold has been reported to be lower, especially in the quadriceps, in patients with PFP in comparison to the healthy individuals [10]. Quality of life (QOL) is disturbed among patients with PFP. Patellofemoral pain can also affect mental health status QOL in patients [11, 12]. These patients may experience disability and limited functional activity [13].

Interventional programs such as core training, whole body vibration, mobilization with movement, and taping are used in patients with PFP [14–19]. A new method in rehabilitation is virtual reality training (VRT). Based on previous studies, VRT is effective in the treatment of vestibular disorders [20], balance impairment [21–24], cerebral palsy [25], pain [26–28], knee osteoarthritis [29], knee arthroplasty [30], and hyper kyphosis [31]. In addition, VRT can improve the functional outcomes and cortical reorganization in stroke, cerebral palsy, and among healthy people [32–36]. These studies showed that VRT affected the prefrontal cortex and caused neuroplasticity in the brain, which resulted in improving motor, cognitive and executive functions. On the other hand, studies that investigated one session of gaming showed that VRT could change the brain activity, such as increased theta wave power in the healthy adult in the frontal cortex that is important in attention and executive function [36], beta power enhancement in the older adult that is important in arousal and cognitive processing [37], and alteration in the activity of temporal and parietal cortex in young people, being related to balance function [38, 39]. These brain changes cause improvement in motor and cognitive functions. Even though there is some evidence on the impact of VRT in the rehabilitation of musculoskeletal impairments, so far, no research has been conducted on the effectiveness of VRT in the management of women with PFP. In addition, studies that investigated the effects of all types of treatment methods on the management of PFP did not pay attention to central changes and the effects of these methods on brain changes at all. Furthermore, given the fact that VRT can affect the brain in neurological patients and healthy subjects, and also brain’s waves power can change during VRT, it seems interesting to study its effects on the brain of people with musculoskeletal impairments. Particularly, the effect of VRT on patients with PFP has not been investigated yet.

Routine management of PFP has focused only on the physical symptoms. We hypothesized that VRT could affect physical symptoms and brain activity together. Therefore, the aim of this study was to make an attempt to improve the performance of patients with PFP by using VRT and to investigate the effectiveness of this method on brain function alterations. In detail, the main hypotheses of this study were hence to investigate whether (1) VRT could improve balance performance, pain level, QOL, and function in patients with PFP and; (2) rather than solely considering clinical effects of VRT, the effect of this program as a new rehabilitation method, on central nervous system and QEEG neurodynamics in patients with PFP was also targeted.

## Methods

This study was a parallel, randomized clinical trial conducted in Rehabilitation Sciences Research Center, Shiraz University of Medical Sciences. A computerized random number generator (www.randomization.com) was used to establish randomization lists before the initiation of the study. In order to blind the assessor to the group assignment, a research assistant prepared sealed, opaque and numbered envelopes containing the participants’ group allocation. Another investigator opened the randomization envelope which indicated the patients’ group assignment. Group assignment was done accidentally with block randomization (13 blocks, 2 in blocks). A blinded assessor, who was not involved in the intervention process, performed the assessments for 3 times (baseline, post-intervention and follow-up). The study was approved by the Ethics committee of research in Shiraz University of Medical Sciences, Shiraz, Iran, (IR.SUMS.REHAB.REC.1398.027) and registered in the Iranian Registry of Clinical Trials (IRCT20090831002391N40, 23 / 10 / 2019).

Participants were recruited to the study through advertisements in medical centers and rehabilitation clinics. Those fulfilling the eligibility criteria were invited to participate in the study, and volunteers were enrolled after signing written informed consent.

The inclusion criteria were: 1) 18–40 year old women with anterior knee pain more than 6 months; 2) onset of pain without the history of trauma; 3) pain during touching patellar facet; 4) 3 or more pain scores on Visual Analogues Scale (VAS) during stepping up and down a 25 cm step or squatting; 5) pain in at least two of this activity: prolonged sitting with knee bending, stairs climbing, squatting, running, kneeling, hopping/jumping [40]; 6) Kujala score lower than 83 [41]; 7) no visual or neurological impairments; and 8) right handedness [42].

The exclusion criteria were included as follows: knee osteoarthritis; history of surgery of the knee or patellar dislocation; history of the laxity of the ligament; patellar tendon pathology; abnormal situations such as leg length discrepancy of more than 2 cm; physiotherapy or acupuncture in the last 3 months; pregnancy; and drug use that affects the balance during the past 72 h.

Calculation of the sample size was according to one of our previous related studies [17], in which balance of patients with PFP improved based on the modified star excursion balance test (mSEBT) measurement after 4 weeks of core muscle strengthening. By considering alpha = 0.01 and beta = 0.8, the minimum sample size was estimated 11 individuals per group. Considering the estimated attrition rate of 20%, the required sample size was 26 patients (13 participants in each group). Participants were assigned into two groups, control and intervention.

### Outcome measures

Prior to the initial assessment, each participant’s information, such as demographic characteristics (age, height, and weight) (Table 1), was collected by the investigator. Aims and processes of the research were described for all participants before starting the trial. The trial outcomes were assessed at the beginning, at the end of the 8 weeks of training, and at the one-month follow-up period. Follow-up was performed in both study groups only on clinical indices. All training sessions were performed under the supervision of a physiotherapist.

**Table 1:** Demographic data of participants and baseline values for pain, star excursion balance test, Kujala, SF-36, and step-down test

		Mean ± SD	Minimum	Maximum	*P*-value^*^
Age (years)	Intervention group (*n* = 13)	29.69 ± 5.69	19	40	0.35
	Control group (*n* = 13)	31.76 ± 5.52	25	40	
Height (cm)	Intervention group (*n* = 13)	162.84 ± 5.20	153	170	0.64
	Control group (*n* = 13)	165.53 ± 5.22	158	176	
Weight (kg)	Intervention group (*n* = 13)	69.30 ± 13.69	47	103	0.58
	Control group (*n* = 13)	66.61 ± 10.70	53	88	
Body mass index	Intervention group (*n* = 13)	26.09 ± 4.68	18.59	35.64	0.27
	Control group (*n* = 13)	24.27 ± 3.48	20.57	32.27	
Pain (mm)	Intervention group (*n* = 13)	54.84 ± 15.31	33	70	0.20
	Control group (*n* = 13)	47.53 ± 13.24	30	70	
Kujala	Intervention group (*n* = 13)	62.38 ± 12.23	28	73	0.33
	Control group (*n* = 13)	66.61 ± 9.43	52	82	
SF-36	Intervention group (*n* = 13)	46.76 ± 14.96	27	75	0.19
	Control group (*n* = 13)	53.92 ± 12.39	34	79	
Anterior SEBT (%)	Intervention group (*n* = 13)	71.37 ± 13.58	52.69	102.03	0.43
	Control group (*n* = 13)	74.76 ± 7.16	65.62	88.24	
Medial SEBT (%)	Intervention group (*n* = 13)	84.78 ± 15.41	48.78	112.59	0.19
	Control group (*n* = 13)	91.76 ± 10.79	75.47	107.77	
Lateral SEBT (%)	Intervention group (*n* = 13)	73.81 ± 11.83	59.17	98.06	0.49
	Control group (*n* = 13)	76.72 ± 9.64	65.44	92.03	
Step down (steps/30s)	Intervention group (*n* = 13)	9.07 ± 5.39	0	19	0.84
	Control group (*n* = 13)	8.69 ± 4.73	2	16	

*SD* Standard deviation, *SEBT* Star Excursion Balance Test; *P-value was significant at the 0.05 level

Balance was evaluated by mSEBT as the primary outcome. Modified SEBT measures balance in 3 directions (anterior, posteromedial, and posterolateral) [43]. By using 3 tapes, a Y-shape was drawn on the floor. The angle between the anterior tape and two others was 135 degrees, and between the two posterior tapes, there was an angle of 90 degrees. Six trials were done before the main trials for familiarity; then, 3 main trials were done and the maximum reach was recorded. The individual’s score was normalized to the leg length. Distance between the anterior superior iliac spine and medial malleolus was measured in supine positions for the acquisition of the leg length [44]. Star excursion balance test is a valid and reliable test [45].

Secondary exploratory outcome measures were pain, QOL, function, and brain activity, which were assessed using VAS, SF-36 questionnaire, Kujala questionnaire and step-down test, and quantitative EEG, respectively. VAS is a line with 100 mm length in which 0 indicates no pain and 100 means extreme pain. Validity and reliability of VAS are confirmed in patients with PFP [46]. SF-36 is a general tool for the assessment of QOL among people. It contains two summary components including the physical and mental component scales. Also, SF-36 has 8 items covering functional status, wellbeing, and overall evaluation of health. A score near 0 indicates a lower QOL. The Persian version of this questionnaire is reliable and valid [47]. Kujala patellofemoral scale is a specific tool with 13 items, and its Persian version has acceptable reliability and validity [48]. For a step-down test, we used a step with 8 in. height, and participants stood on it, stepped down and returned with non-test lower extremity; the examiner counted the numbers of contact between the heel and floor in 30 s, and 3 trials were done. Maximum of correct repetitions was considered as a person’s score. This is a reliable test in PFP [49].

### EEG recording

Quantitative electroencephalogram (NR-Sign EEG 3840 device, NR-Sign Inc., Vancouver, Canada) was used for recording the electrical activity of the brain cortex. The linked ear served as the reference point. Prior to recording, conductive gel was applied in the sites of electrodes to reduce the impedance of skin below 5 Ω. Silver-chloride surface electrodes were used for signal recording (Medico Electrodes International Ltd., Uttar Pradesh, India). According to 10/20 system, 24 electrodes (Fp1, Fp2, F7, F8, F3, F4, T7, T8, T3, T4, P7, P8, P3, P4, FC3, FC4, C1, C2, Fz, Cz, Pz, Oz, FCz, CPz,) were fixed on the scalp. Participants were asked to stand on their right leg on a stable surface with eyes opened, and then EEG was recorded for 3 min. Before the main record, 2 familiarity trials were done. Band pass filter was 2–120 Hz and data were collected at 500 Hz. EEG recording was done in School of Advanced Medical Sciences and Technologies.

Neuroguide Software (NG 2.5.5; Applied Neuroscience, St Petersburg, FL, USA) was used for EEG data processing. An EEG expert preprocessed the recorded signals by means of achievement of denoised signal (to eliminate signals disturbed by eye movements, motion or muscle artifacts). Automatic editing was done using Neuroguide software. According to Neuroguide Software QEEG normative value database, the difference with the Z-score power was computed by Fast Fourier Transformation.

### Intervention

No interventions were done in the control group and they continued their usual and normal lifestyle. We provided them with the necessary training educations to manage daily programs and activity daily livings regarding PFP. These educations were provided to participants as a prepared text that contained information about how to manage daily activities such as preventing prolonged sitting, controlling the frequency of using of stairs, using proper shoes, not being in a certain knee position for a long time, and not bending the knee for a long time. To control the education in this group, the therapist communicated to the participants via text message. Rehabilitation program was implemented after the end of the study.

The intervention group, in addition to educations provided to the control group, received 24 sessions within 8 weeks with XBOX Kinect™ 360 (Microsoft, Redmond, WA, USA). Exercises in the intervention group were presented 3 times a week. The games used in this study are from three packages: Kinect Adventures, Kinect Sport, and Your Shape Fitness Evolved 2012. The intensity of the games was from easy to hard and based on the ability and progress of each patient in doing each step of the game. Each training session lasted about 40 min, in which the person performed 5 min of warm-up before the game, 5 min of cooling after the game, and 30 min of practice with XBOX. The participants could play freely and move their whole body in the space with Kinect. Previous studies reported the effectiveness of Kinect Adventures and Kinect Sport in the improvement of postural control [50, 51]. Your Shape Fitness Evolved 2012 is a game that consists of strengthening program and a game like tai chi. Squatting, lunging, single leg stance, jumping, simultaneous upper, and lower body movement are the most important movements in this game.

### Statistical analysis

Data were analyzed using SPSS for Windows 16.0 (SPSS Inc., Chicago, IL, USA). The Kolmogorov-Smirnov test was used to check the normal distribution of data. Since the results confirmed the normal distribution of all variables, parametric tests were used for statistical analyses. Repeated measures analysis of variance (ANOVA) was conducted to compare pain, balance, QOL, and function at different times (pre-intervention, post-intervention, and follow-up); a t-test was conducted to compare QEEG data at pre- and post-intervention. *P*-values less than 0.05 were considered significant.

## Results

**Fig. 1:**
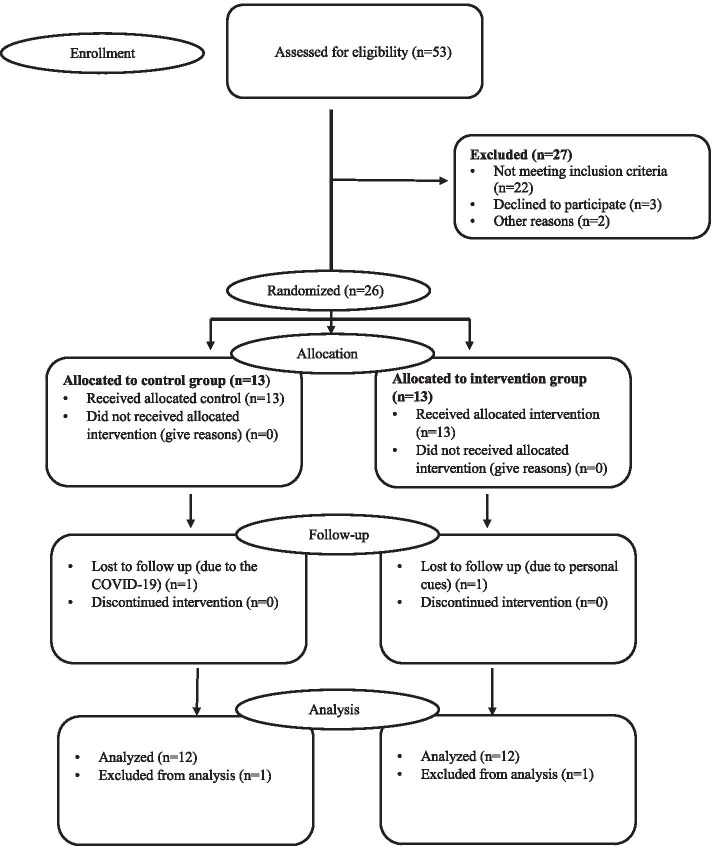
CONSORT flow chart

Fifty three volunteers were assessed for participation in the study. Twenty-seven (50.9%) patients did not meet the inclusion criteria or were excluded according to the exclusion criteria, as they have knee osteoarthritis, age more than 40 years old, left handedness, … (Fig. 1). Twenty-six right-handed women with PFP participated in the present study. Two patients, one due to Covid-19 and the other one because of a personal issue, did not complete the follow-up. The attrition rate was nearly 8%. No side effect was seen after VRT among participants. This study, conducted between November 1st and March 20th, 2020.

The mean age of participants was between 29 to 32 years old. There were no significant differences in age, weight, and height between the two groups. Also, baseline assessment showed no significant differences in clinical parameters (mSEBT as primary outcome; pain, QOL, function, and brain activity as secondary outcomes) between the two groups (Table 1).

Consistent with the primary hypothesis, VRT improved clinical symptoms of patients with PFP, because repeated-measures analyses revealed a significant measures×group interaction for anterior mSEBT (*F*_*2, 24*_ *= 43.31, P < 0.001*), medial mSEBT (*F*_*2, 24*_ *= 45.12, P < 0.001*), lateral mSEBT (*F*_*2, 24*_ *= 22.50, P < 0.001*), pain (*F*_*2, 24*_ *= 6.13, P = 0.004*), Kujala (*F*_*2, 24*_ *= 18.68, P < 0.001*), and SF-36 (*F*_*2, 24*_ *= 8.70, P = 0.001*).

**Fig. 2:**
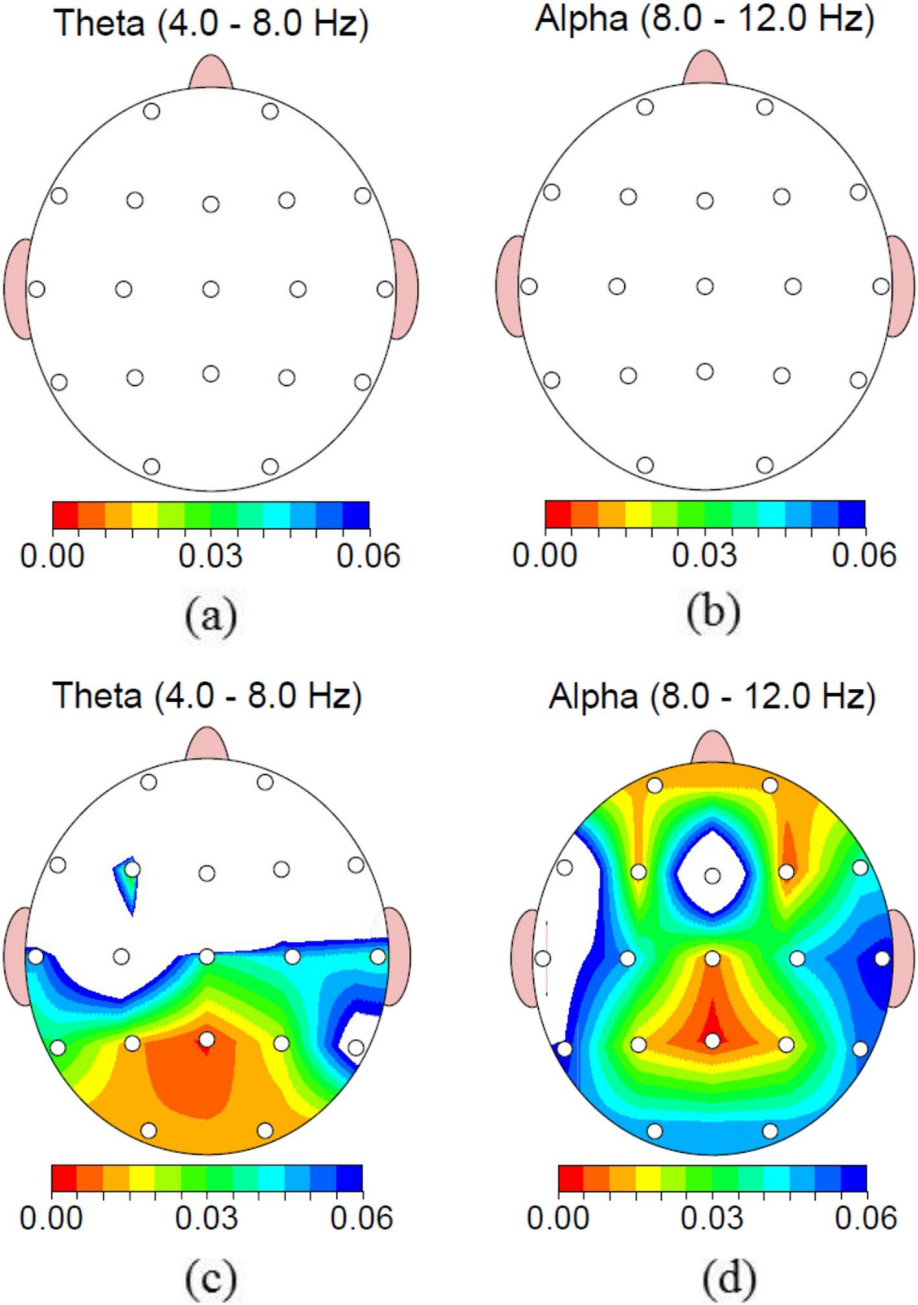
The *p*-value of pre/post VRT group and control group alpha and theta changes (FFT power group independent t-test). **a** Pre-test theta **b** Pre-test alpha **c** Post-test theta **d** Post-test alpha. Colors indicate the significance level (Z-score less than − 1.2 or more than + 1.2)

Because there were only two groups, there was no need for post hoc test and SPPS errors for post hoc test. Figure 2 reports brain mapping of the *p*-value of between group analysis in two times of assessments for alpha and theta power. In response to second hypothesis, after the intervention, alpha (frontal *P = 0.01*, parietal *P = 0.01,* occipital *P = 0.05*) and theta (frontal, parietal and occipital *P = 0.01*) power increased significantly in the VR group in comparison to the control group. Colored area indicates significant changes. Blue colors mean stronger significancy. According to this figure, at the baseline, there were no significant differences between the two groups about alpha and theta power. However, at the end of the intervention, alpha and theta power changed significantly between the two groups.

## Discussion

The changes in pain, balance, function, and QOL after 8 weeks of VRT were assessed in patients with PFP. VRT was associated with significant improvements in these clinical indices. These findings suggest that VRT can help the patients to manage their PFP and its related impairments.

According to the results of the current study, VR group showed balance improvement. Other secondary outcomes, including function, QOL, pain, and brain mapping, also showed significant improvement after using VRT, when compared to the no-exercise group.

Improvement of balance needs motor learning process which relies on feedback and practice [21]. These items are two key components of VRT [52], so when a person plays video games, it can accelerate the motor learning process which causes postural stability enhancement [53]. Besides, in conventional exercise therapy when patients do an exercise repeatedly, they may feel exhausted and may not continue exercising, but at VRT, patients do an exercise repeatedly without any complaint or exhaustion because this type of training motivates them, and they do an exercise with interest. On the other hand, exercises such as single leg stance, single leg stance with arms moving, narrow stance squatting, lunging, and wide stance squatting are routine exercises for promotion of balance [54]. These types of exercises were repeatedly made by our patients during the VRT program, especially in Kinect Adventures and Kinect Sport games. In fact, in the current study VR exercises were selected based on balance, strengthening, and stretching programs. Your Shape Fitness Evolved 2012 that has tai chi-like exercises and tai chi is a training that mix the core muscle training with lower extremity exercises; 3 main positions in these exercises are squat, lunge and plie. These exercises cause loading on the lower extremity and core muscles, so they can result in muscle strengthening and improvement of balance, cognitive, and coordinative skills [55].

There was no research that investigated the effect of VRT on PFP, but some previous studies have investigated using VRT among older adults, after ACL reconstruction, low back pain, and healthy adults. These studies have reported that VRT can improve balance [23, 56–59]. Our results support the findings of these studies. However, our findings contradict the findings of other studies which have reported that VRT was improved leg muscle strength and function but could not cause any improvement in the balance among patients with total knee replacement and older adults [30, 60]. The discrepancy between these two studies and our findings may be mainly due to different samples and differences in the volume, duration, and type of VR games used in these studies. Balance changes in the intervention group were clinically significant because MDC for all 3 directions of mSEBT was reported between 8.7 to 11.5 cm [61], whereas in the VR group mSEBT was changed > 18 cm in all directions at post-treatment and follow-up assessment.

VRT causes an increase in alpha and theta power in the frontal, parietal, and occipital lobe. This is in line with Peterson et.al. that investigated short term balance training with VR among healthy adults. They reported that theta enhancement may indicate an improvement of sensorimotor processing [42]. Besides, theta increase was in line with that in Anguera et al.’s study; they showed 4 weeks of VRT caused theta enhancement in the frontal area among older adults [62]. This similarity in the results of the two studies may reveal enhancement of cognitive control which is the results of VRT. Liu et al. also reported that tai chi led to an increase in theta power in women [63]. The increase in the Theta power is also related to an increase of relaxations, error detection, and attempt to protect postural stability, attention and concentration [64–66]. Therefore, theta improvement may indicate more error detection and enhancement of balance processing that resulted in an improvement of postural stability.

Alpha power enhancement is correlated with automaticity; after the learning process, a task needs lower active cortical processing, and lower attention causes the alpha power to increase [67]. Alpha increase in the VRT group may indicate that, because of practice, they can do a task more skillfully and they can deeply concentrate on their movements, so this affects EEG recording and relaxation state is seen in EEG. Alpha activity that appears in the frontal and central region in addition to the occipital area revealed improvement of the concentration capacity of the mind in the VR group [63]. It means, patients are more stable and do balance tasks more accurately and with less effort, so learning is done and balance performance is improved. This may be a plastic change in the brain. Burcal et al. investigated the effect of balance training in motor planning among patients with chronic ankle instability. After 4 weeks of intervention, there was no change in cortical motor planning [68]. Our result is in contrast with those of Schattin et.al. that showed a reduction in theta power in the prefrontal cortex after 8–10 weeks of video game training in older adults [69]. This could be related to differences in the types of intervention and also the position where the EEG was recorded. On the other hand, Burcal’s study had a small sample size and probably with a higher sample size result could have changed. In Burcal’s study, routine balance training had no effect on EEG, and in Schattin’s study exergame was more effective than routine balance training on EEG. This may reveal the priority of VRT to conventional balance training for brain plasticity. Other reasons for this conflict may be Tai chi-like exercises. Previous studies reported that Tai chi led to an increase in theta activity and resulted in relaxation [70]. In addition, tai chi is effective on the cognitive function and balance control [71]. Thus, it seems that tai chi with virtual reality mode leads to plastic change and alters the neural function in the brain (especially in the prefrontal cortex and parietal cortex), and this corresponds to an improvement in functions, such as postural stability.

Our results are consistent with previous studies in that VRT is effective in reducing pain [59, 72]. Reduction of pain in the VR group exceeded the minimal detectable change (MDC) for VAS which is 1.5 to 2 cm in patients with PFP [46], whereas the average improvement in pain after VR training was more than 2 cm and it was maintained until the 1 month follow-up. This indicates that improvement of pain is clinically meaningful for patients who have used VRT.

One reason of pain reduction after VRT may related to neurodynamics changes of the brain. Alpha therapy is a method for pain reduction. Alpha therapy accompanied by relaxation and distraction is more effective on pain relief [73]. In our VRT, relaxation was induced by tai chi like exercises. There is a cycle between pain, arousal, anxiety and relaxation; relaxation causes reducing sensory input which resulted in reduction of the level of arousal, arousal reduction causes reduction of anxiety and in final, this anxiety lowering resulted in lower pain perception [73]. Distraction was a key factor in VRT and is a specific characteristic of virtual reality environments. So, these reason may affect pain reduction. Ahn et al. investigated the effect of transcranial alternating current stimulation on alpha oscillations in patients with chronic low back pain. They reported that enhancement of alpha oscillation with transcranial alternating current stimulation causes pain relief in patients with low back pain [74]. So, it is possible that VRT induced pain reduction by enhancement of alpha in patients with PFP.

Change of QEEG neurodynamics and its indicators after VRT may be because of some mechanisms such as neuroplasticity, cortical reorganization, and motor learning. These mechanisms can be happened due to activation of mirror neurons and also activation of neural pathways which were inactive and not utilized before treatment. The other reason may be practice dependent enhancement. When patients do an exercise repeated and repeated, neuroplasticity can be induced [32, 33].

Reduction of pain and improvement of balance may cause an improvement in QOL and function of patients. Minimally important change for Kujala questionnaire was reported about 10 points. Improvement of the Kujala questionnaire in VRT was more than 16 points in the post-treatment and follow-up, so this change is clinically significant. In the control group, the scores of this questionnaire did not change significantly. Physical and mental issues are very important in the health-related QOL. Anxiety and mood problems are also strongly associated with health-related QOL [75]. Our findings that reveal VRT can improve physical function and pain; also, it can alter the brain activity and cause a relaxation state and reduced anxiety, so VRT results in improvement of the health-related QOL.

Based on the above points, VRT can be added to routine physical therapy interventions for PFP. This is a new method that not only improves the clinical symptoms of PFP (such as pain, balance, QOL, and function), but can also lead to good results and beneficial changes in the brain. VRT can be considered as a holistic approach in the rehabilitation of PFP. Our results also corroborate with the efficacy of VR in the treatment of musculoskeletal impairments. The findings of this study may be used as evidence to inform therapists and specialists in promoting a holistic intervention for PFP rehabilitation. VRT can be used in clinical and sports medicine for encouraging patients to treatment and motivate them to continue their intervention period. We suggest that in the future studies comparisons between routine balance training and VRT would be assessed in PFP and their brain functions. Therefore, it is recommended that in future investigation more detailed mental and psychological effects of VRT for PFP rehabilitation should be investigated.

### Study limitations

Our participants were women, the reason was that PFP is more prevalent in women and sex is an effective and important underlying factor in PFP, so our findings could not be generalized to all male individuals with PFP. Second, we didn’t record QEEG in follow-up period. Recording QEEG in follow-up could provide information about the durability of brain changes. Another limitation was the time of the follow-up stage, because there was no similar study, determining an exact effective follow-up period was not possible; however, this could be regarded as a basic efficient time for similar future studies.

## Conclusion

VRT is a good and new conjunct treatment for PFP. We suggest that future studies compare routine balance training and VRT in patients with PFP and record their brain functions with QEEG and fMRI, also this method can be investigated among other musculoskeletal disorders.**”**

## Data Availability

The data file of this study is available to the corresponding author and can be made available to anyone upon reasonable request.

## Abbreviations

VRT: Virtual reality training
PFP: Patellofemoral pain
QOL: Quality of life
VAS: Visual analogue scale
mSEBT: Modified star excursion balance test
MDC: Minimal detectable change
